# Media content analysis of the introduction of a “soft opt‐out” system of organ donation in Wales 2015‐17

**DOI:** 10.1111/hex.12872

**Published:** 2019-02-06

**Authors:** David J. Dallimore, Leah McLaughlin, Carol Williams, Jane Noyes

**Affiliations:** ^1^ School of Social Sciences Bangor University Bangor UK

**Keywords:** deemed consent, media content analysis, organ donation, soft opt‐out, Wales

## Abstract

In an attempt to improve organ donation rates, some countries are considering moving from “opt‐in” systems where citizens must express their willingness to be an organ donor, to “opt‐out” systems where consent is presumed unless individuals have expressed their wishes otherwise, by, for example, joining an “opt‐out” register. In Wales—a part of the United Kingdom—the devolved government recently legislated for an “opt‐out” system. For the change to be effective, a public awareness campaign was critical to the policy's success. Using quantitative and qualitative content analysis, we explored media coverage of the change to better understand the relationship between the state, policy actors, media and the public when such policy changes take place. Our findings illustrate how a state communication campaign can effectively set the media agenda within which we saw a degree of interdependency created with the state using the media to promote policy, and the media relying on the state for credible information. Yet we also found that the media is not uncritical and observed how it uses its autonomy to influence policy setting. Over the period of study, we found that a change in tone and view towards deemed consent organ donation has taken place in the media. However, while this may influence or reflect public attitudes, it is yet to be seen whether the media campaign translates into behavioural change that will result in increases in organ donations.

## 1 INTRODUCTION

In 2015/16, there were more organ transplants in the United Kingdom (UK) than ever, but despite increases the UK has one of the lowest rates of consent to deceased organ donation in Europe.^1^ Targeted activity focusing on changing attitudes and behaviour has been taking place across the UK, but in 2015 the Government in Wales (which has devolved powers from the UK Government including responsibility for health) became the first UK country to introduce an “opt‐out” system for consent to deceased organ and tissue donation through the Human Transplantation (Wales) Act 2013. Prior to this, an opt‐in system of consent was in place. The new Welsh legislation provides an “opt‐out” default where unless a person has explicitly registered or expressed their decision to “opt‐out” of being an organ donor during their lifetime, all residents of Wales who are over 18 years with mental capacity are presumed to have given their consent to deceased organ donation. This is also called “deemed consent.” It is however a “soft opt‐out” system where family members are involved and asked to support the deceased persons decision made in life, whether it was registered on the organ donor register, expressed verbally or deemed (as opposed to a “hard opt‐out” where families are not consulted). The legislation came into force on 1 December 2015 and aimed to increase the number of organ donations in Wales and save more lives through organ transplants and is part of a broader strategy to increase UK consent rates to 80% by 2020.^2^

In order to be successful, the Act and the associated media campaign needed to inform the public of the change, create a situation whereby organ donation becomes the norm, and create a way for people to behave as the Act intended.^3^ As one of only a small number of countries world‐wide to have introduced a presumed consent system, the legislation constitutes a major change to the partnership and social contract between the Welsh Government and the people of Wales.^3^ The Act is inevitably controversial and not everyone consulted agreed with the “soft opt‐out” system and its principles. Some (eg, Ref. 4) warned that an “opt‐out” system would result in a backlash from members of the public who currently support organ donation, which could have a negative impact on donation rates. Others (eg, Ref. 5) argue that only a change to an “opt‐out” system could improve UK organ donation rates and accordingly save lives, a conclusion supported by research that has modelled donor regimes.^6^ Such arguments for and against were played out in political and public debates and across the media and continue to do so.

In this paper, we focus on this changing discourse around organ donation within the confines of the introduction of a “soft opt‐out” system and in doing so examine the relationship between state policies, the media and public opinion. We review previous literature from studies of the media and its influence on organ donation then summarize the debates around public opinion, the media and the role of the state in public policy. We present the methods, then report and discuss our findings. In our analysis, we found close intertextuality and interdependence between the state and the media, observing that the health‐related messages successfully framed by state organizations to promote their policies are used by the media to create human interest stories that appeal to readerships. We conclude that over the period of study, there has been a change of tone in media coverage towards support for the new organ donation policy, a change that is independent of irrefutable evidence that the policy is effective in Wales.

## 2 BACKGROUND

### 2.1 Media and organ donation

A body of literature exists examining the influence of different sources of information on attitudes towards organ donation. Matesanz and Miranda^7^ identify mass media as having potentially positive and negative effects on public attitudes. They highlight historic moments when mass media was observed to have had a significant, if short‐term impact on organ donation rates. For example, in 1980 after a prime‐time UK current affairs programme questioned the validity of brain death criteria (Panorama, BBC) it took 15 months for donor referral rates in the UK to recover. Conversely, the effect of increased organ donation seen in Italy following a highly publicized shooting of 7‐year‐old child and subsequent donation of his organs was seen to have a highly positive effect on public opinion.^7^ A number of studies^8^, ^9^ have shown that the media is a primary source of information about organ donation—although based on research from the pre‐mass‐Internet age, the common findings that television has the greatest impact will inevitably be outdated. Nonetheless, these studies are helpful in consistently identifying specific types of communication that tend to have favourable or non‐favourable effects on public opinion towards organ donation. Negative opinions have been shown to stem from mass media while positive attitudes are more likely to come from information given by health professionals and from discussions with family and friends.^8^, ^10^

The effect on actual transplantation rates was shown to be positively impacted by mass media in a content analysis of US television news over the period 1990‐2005.^11^ However, other research suggests that coverage in television dramas and documentaries along with sensationalist press coverage can have more negative impact, including the development of myths about black markets in organs, corruption within the medical community and preferential treatment of certain groups or individuals for transplant.^10^, ^12^ While studies have examined the influence of media on public attitudes towards organ donation and on organ donation itself, the relationship between public policy or legislation, the media, and public opinion has not been widely examined. This study aims to fill that gap and respond to previous studies examining presumed (also known as deemed) consent systems (eg, Ref. 13) where contextual evidence, such as media content, is highlighted as missing from debates.

### 2.2 Media, policy and public opinion

Within the policy studies literature, media institutions are acknowledged as purveyors of information, as ciphers for competing ideas, and in some cases, policy agenda setting.^14^ Actors within the policy process assume that media is a pervasive influence—whether it is or not—and success is often measured by the extent of media coverage of any particular agenda.^15^, ^16^ The media campaign around the Human Transplantation (Wales) Act 2013 was seen as vital to its success. For the policy to work, people in Wales had to have heard about the changes. The campaign was run by the Welsh Government and National Health Service Blood and Transplant for two years preceding full implementation on 1 December 2015 and continued following the implementation period. The media coverage and tone at this time were a key point of interest to examine and to evaluate what happened over these critical years and better understand the complex relationship between health policy, the media and public opinion.

Study of mass media content is often used to decipher the relationship between media and public opinion. It has long been seen as a means of monitoring the “cultural temperature” of society either from a humanist perspective in looking back to identify what it says about society and the culture producing it, or a behaviourist approach, looking forward to the effects that content produces.^16^ This reflects debates about whether mass media *creates* public opinion, attitudes or perceptions, or *reflects* existing attitudes perceptions and culture^17^ or as most scholars agree, does both.

When considering public policy however, the relationship between the state and mass media organizations needs to be examined to establish the antecedents of these constructions. In media studies, this relationship has often been assessed according to a propaganda model^18^ contending that the state exerts a powerful influence over media through regulation, through censorship, and as a major source of “reliable information.” The state relies on the media for the communication and legitimizing of its policies, and maintaining the status quo.^19^ This dynamic influences what is emphasized and what is absent in media reporting through what Gramsci^20^ refers to as the “negotiation of hegemony.” The opposing view is that the influence of the state over the media is not absolute.^19^ Hall^21^ argues that mass media organizations have a degree of autonomy through which they can differentiate their views from the state and exert influence on politics and society independently as both drivers and reflectors of public opinion.

The media can also be seen as operating in at least two interlinked ways regarding public policy.^15^ First, media influences the policy process by playing a gatekeeping role in whom is given status to comment on public problems and prescribe solutions. Journalists decide which policy actors (individuals or group that are directly or indirectly, formally or informally, affiliated with or affected by the policy process at any stage) are taken seriously as important players ascribing them with a particular “standing.” Secondly, journalists and editors are more than gatekeepers in allowing policy actors a mouthpiece, but are themselves players in the policy process, shaping and framing the discussion. Media “frames” the ways in which issues are organized and understood in the public realm by organizing words, images and themes that are used to introduce and present a public policy issue. Frames are “a necessary property of a text—where text is broadly conceived to include discourses, patterned behaviour, and systems of meaning, policy logics, constitutional principles, and deep cultural narratives”.^22^ ^, p. 37^

In this study, we are interested in using text frames to examine who says what; through which channels; to whom; and most importantly, to what effect over the period of time since “opt‐out” consent to organ donation legislation in Wales was first conceived, across the implementation phase (2015‐2017). In presenting evidence, we therefore examine the relationship between policy actors, the state, the media and the public, to gain understanding of the dynamics of interrelation, assessing the extent to which mass media sets or reflects public policy agendas.^23^ We draw upon Shoemaker and Reese's theoretical approach,^16^ which focuses less on the *process* and *effects *of how media messages are given and received by audiences, but encourages us to investigate the factors *inside* and *outside* media organizations that affect content, incorporating the following objectives:

- Review media coverage of organ donation prior, and post‐implementation of the Human Transplantation (Wales) Act 2013.
- Examine relevant media content with foci on their newsworthiness, the framing of messages and the internal and external drivers of content.
- Investigate the factors which influence media content, and therein the relationship between the media, public policy and public attitudes.

## 3 METHODOLOGY

We used what can be described as a “summative” content analysis^24^ where we start by identifying and quantifying particular words and content within the text with the purpose of understanding the contextual use of the words or content. We categorized the media message related to soft opt‐out organ donation for valance^25^ (positive, neutral, negative tone) and assessed its newsworthiness through the twin dimensions of deviance and social significance.^26^ We then counted the frequency of news stories across time periods. Finally, we qualitatively explored the discourses within the narrative messages that the stories contained. This facilitated investigation of meaning by bridging the gap between texts and readers’ interpretations of them.^27^ We were therefore able to examine the preference given to some arguments by journalists and look for sources and the explanatory frameworks which underpin them.^28^

### 3.1 Sampling

A purposeful approach was taken in selecting all articles from key media. This consisted of news texts published either online, or available online that refer to organ donation policy in Wales during the period January 2015 to October 2017. Search terms deployed included “Wales” AND “Human Transplantation” OR “Transplant” OR “Organ Donation” OR “Organ Donor.” A focus on textual material was made as time and resources forbade analysis audio or video sources. This is an acknowledged short‐coming of the investigation, although many written reports mirrored or reported on audio and video sources, which mitigates the potential impact of this limitation. The dates were chosen to include coverage of the Act from its implementation in 2015, thereby excluding speculative discussion that preceded it. Within the sample are news and opinion pieces, as well as public reaction to them in the form of user‐generated comments. The importance of the latter in shaping or reflecting public policy has grown in importance as the boundaries between “old” and “new” media become less clear and the public become both consumers and producers of content.^29^ However, some research^30^ has cast doubt on user‐generated commentary as representative of public opinion—which it is clearly not—and its influence. While it has been shown to be widely read and popular, user content is also found to be treated very sceptically by the public.^31^, ^32^ In this study, a systematic search of online news sources was undertaken and focused on obtaining relevant findings for the objectives of the study and within the parameters set out above. Sixty items were identified, and texts were imported into NVivo (v.11) software for analysis (Table 1).

**Table 1:** Media sources

Headline	Publication	Publication date	Influence weighting
Organ donation crisis threatens hundreds of lives	The Telegraph	19/01/2015	22
Organ opt‐out law campaign targets workplaces	BBC Online News	19/01/2015	30
Should the UK change the organ donation system	Blasting News	27/03/2015	3
Opt‐out' organ donation register launched for the first time—ahead of Welsh presumed consent law	Wales Online	09/07/2015	11
What will you choose to do—Wales' new organ donor register launches	ITV News	09/07/2015	15
Organ donation law awareness campaign “huge challenge”	BBC Online News	23/08/2015	30
Church responds to organ donor law change	Anglican News Service	18/11/2015	1
Organ donation—does presumed consent work?	The Conversation	19/11/2015	4
Rhys Meirion urges families to discuss their organ donation wishes with each other	Daily Post	22/11/2015	7
New opt‐out system in Wales aims to revolutionise organ donation	The Guardian	25/11/2015	24
Organ donation law “revolution” starts in Wales	BBC Online News	01/12/2015	30
Wales switches to organ donation opt‐out	The Guardian	01/12/2015	24
Organs can be taken from the dead	Mail Online	01/12/2015	23
Families veto hundreds of organ donations in 5 years, figures show	The Guardian	15/01/2016	24
Organ donations vetoed by hundreds of bereaved families	BBC Online News	15/01/2016	30
Awareness of organ donation change in Wales rises	BBC Online News	01/02/2016	30
Three‐quarters of Welsh adults are now aware of new organ donation system	Wales Online	01/02/2016	11
Lara's legacy—Widening the search for a “genetic twin”	BBC Online News	09/02/2016	30
Tattoo on murdered teen's arm persuaded his mum to honour organ donor wish after caravan park horror	Mirror Online	14/03/2016	18
First Welsh organ donation statistics released since presumed consent system launch	ITV News	18/03/2016	15
Six of 15 organ donations in Wales done through new opt‐out system	South Wales Argus	18/03/2016	6
Education pack aims to ease shortage of organ donors	Schools Week	19/03/2016	n/a
Harvest organs from living euthanasia patients, Dutch researchers propose	The Catholic Herald	01/04/2016	3
Union leader who's kept alive by BATTERIES backs campaign for opt‐out organ donor register	Mirror Online	03/04/2016	18
Government planning “opt‐out” system for organ donation	The Irish Times	06/04/2016	n/a
Courageous' couple praised for sharing their organ donation story	Cambrian News	10/04/2016	2
Remove organs from euthanasia patients while they're still ALIVE'	Mail Online	01/06/2016	23
Wales organ donations “encouraging” in year after consent law	BBC Online News	01/06/2016	30
Organ donation—Why you should be like Scot and save the lives of others	Express and Star	02/06/2016	9
Selfless Doncaster gran inspires others to join organ donor register	Doncaster Free Press	03/06/2016	4
Welsh parents tight‐lipped over organ donation	ITV News	03/06/2016	15
Campaign launched to get parents talking about organ donation with their kids	Salford Online	05/06/2016	1
Scientists attempting to harvest human organs in pigs create human‐pig embryo	The Guardian	06/06/2016	24
Dozens saved' in 6 mo by Welsh deemed consent organ donation system	The Guardian	14/06/2016	24
Half of organ transplants from deemed consent after new law	BBC Online News	14/06/2016	30
Opt‐out organ donation system saves dozens of lives in its first 6 mo	Daily Post	14/06/2016	7
The numbers that show Wales' “opt‐out” organ donation system is saving lives	Wales Online	14/06/2016	11
Doctors to lobby for opt‐out organ donor system	The Guardian	22/06/2016	24
Wales leading the way in organ donations, says top doctor	BBC Online News	01/09/2016	30
Welsh “deemed consent” organ donation system shows promising results	The Guardian	04/09/2016	24
Every day I think of the selfless gift my donor and their family gave me'	Wales Online	01/12/2016	11
One year since the automatic organ donation law came into force—has it made a difference	ITV News	01/12/2016	15
New Opt‐Out Organ Donation System Is a Good Idea	Science of Us	03/01/2017	10
All French citizens are now organ donors unless they opt out	The Independent	04/01/2017	20
When organ donation isn't a donation	British Medical Journal	28/02/2017	n/a
Organ donation breakthrough promised after new tissue warming discovery	The Telegraph	01/03/2017	22
We shall look at opt‐out law for organ donation	IOM Today	28/03/2017	n/a
A family moved to Wales to increase son's chance of finding an organ donor after a rare disease	Wales Online	31/03/2017	11
Why it's time to introduce “opt‐out” organ donation	The Irish Times	04/04/2017	1
We could make you organ donor by default, says No. 10	Mail Online	01/07/2017	23
Cancer patient had TEN organs removed and six replaced after four‐stone tumour spread through body	Mirror Online	13/08/2017	18
Now I'm able to breathe'—Welsh transplant patients share their experiences of receiving lifesaving donations	Wales Online	02/09/2017	11
Families encouraged to talk about organ donation to “reduce transplant waiting lists”	ITV News	04/09/2017	15
Gwynedd woman's sight saved thanks to double eye transplant from “heroes she never knew”	Daily Post	04/09/2017	7
A man who got a sight‐saving eye transplant wants more people to talk about donating organs	Wales Online	05/09/2017	11
Barrow mum saved by sister's kidney speaks about the importance of organ donation	The Mail	06/09/2017	23
Young people should “have the chat” about organ donation	Wales Online	06/09/2017	11
Brain aneurysm girl helped eight people through organ donation	South Wales Argus	08/09/2017	6
Trade union chief to send letter to family of stranger who saved his life with heart transplant	Mirror Online	08/09/2017	18
Organ donation: Does an opt‐out system increase transplants?	BBC Online News	10/09/2017	30

Weighting calculated from the country website ranking (www.alexa.com) of each news source then ranked by its size relative to the other sources in the list. If more than one source has the same rank, the top rank of that set of values is presented. Higher numbers indicate greater weight of influence.

### 3.2 Data analysis

**Figure 1:**
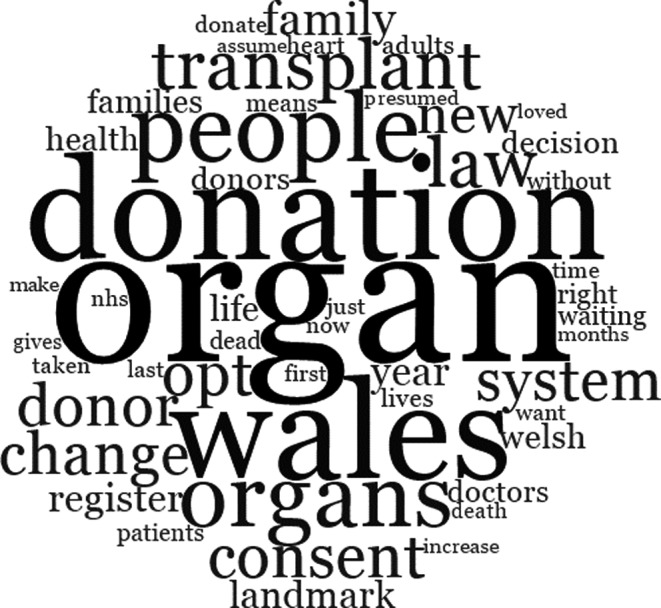
Word frequency

Our analysis began with searches for occurrences of identified words and messages within the sources (Figure 1). While their frequency is interesting, to understand the underlying contexts of messages within the sources further information was needed. As well as the subject‐specific messages, other key content variables were recorded including media weighting to allow high circulation, high rating or highly influential media to be scored higher than small, less important media. This is particularly important in the case of a small country such as Wales where UK‐wide and predominantly London‐based media have been shown to have greater reach and impact than regional, Wales‐based media.^33^ In this study, influence is measured by the country website ranking (www.alexa.com) on which the articles were published with a weighting calculated by taking the percentile rank of the range of news articles collected (ie the percentage of scores in its frequency distribution that are equal to or lower than it). Also recorded were the number of “shares” and where appropriate, the number of user‐defined “comments” although inconsistency in the availability of these variables meant that we did not account for these in our final analysis.

We examined each news item headline and assigned a valance variable^25^ of either positive, negative or neutral to indicate the tenor of reporting of the “soft opt‐out” policy in Wales. In a small number of cases where the headline was ambiguous, it was also necessary to further skim the content. Within content, texts, words and phrases were judged to identify the tone of text towards the coded categories, for example, attitudes expressed editorially or in quotations, towards the concept of “opt‐out” organ donation. These were further coded as either positive, neutral or negative in their tone towards the introduction of the legislation in Wales. We also drew upon Feeley et al’s approach^23^, ^26^ to assessing newsworthiness of articles about organ donation in which the prominence given to the topic is related to the deviance of the news story and the significance of the news event in relation to its social, economic or cultural ramifications. Contextual factors that may affect meanings taken from the text, such as the position and credibility of spokespersons or sources quoted, were noted.^34^ ^, p. 163^

### 3.3 Procedure

Search results (online news articles) were saved as pdf files. The files were skimmed for content not relevant for the research question, such as side‐bar advertisements. Data were imported into the data analysis software and articles were assigned variables using a source classification sheet that included publication, date of publication and UK site ranking. Each news transcript served as the unit of analysis for investigation.

The coding scheme was developed with key concepts highlighted based on the objectives of the study, text‐frequency analysis and an initial review of content (Table 2). Automatic word searches were used to establish incidences of word or phrase use within the data and then manually coded within the schema for valance (positive, negative or neutral), deviance (1 = not at all unusual, 2 = somewhat unusual, 3 = unusual and 4 = extremely unusual) and significance (1 = not at all significant, 2 = minimal significance, 3 = moderate significance, 4 = major significance). Two researchers served as primary coders, providing validity through all of the articles being coded twice. To ensure that each coder achieved a satisfactory level of understanding and agreement, six sample transcripts were selected at the start of the process for discussion to ensure consistency. Statistical measures of inter‐rater reliability showed an average of 95.6% coder agreement and an average Kappa coefficient of 0.58 (which accounts for chance) and are deemed fair to good agreement. Descriptive and inferential statistics were conducted to inductively explore findings and address research questions using IBM SPSS v.22.

**Table 2:** Coding scheme

Concept	Code	Words/phrases
Attitudes towards opt‐out systems	Opt_out	*“opt‐out*,” “opt‐in,” “*presumed consent*,” “*deemed consent”* and synonyms
Relationship between state and citizens	Law_change	*“law,” “legal rights,” “legislation,” “State,” “government”* and synonyms
Discourse around issues of consent	Consent	“*choice*,” “*freedom,” “consent*,” “*wishes,” “decisions” *and synonyms
Organ donation as act of deceased	Donor_Gift	*“altruism,” “gift,” “giving,” “selfless,” “sacrifice” *and synonyms
Recipient perspective of organ donation	Recipient_Gift	*“generous,” “receiving,” “gift of life,” “giving” *and synonyms
The role, and changed role of the family of the deceased	Family_role	*“Family” AND “conversation,” “discussion,” “debate,” “*veto*,” “refusal,” “override”* and synonyms

## 4 RESULTS

When examining each news headline for valance—or tone—towards the Welsh “opt‐out” policy, we found that of the sixty sources analysed, 55% had a positive tone (eg *Automatic organ donor register introduced in Wales to boost transplant numbers*. Daily Mirror, 05/12/15), 15% were negative (eg *Organs can be taken from the dead without prior consent: Landmark law change in Wales*. Mail Online, 01/12/15) and 30% neutral (eg *New opt‐out system in Wales aims to revolutionize organ donation*. The Guardian 25/11/15). When we applied influence weights calculated from the popularity ranking of each news website, a variance was noted (49% positive, 18% negative and 33% neutral), suggesting that highly ranked news providers were more circumspect in their coverage.

**Figure 2:**
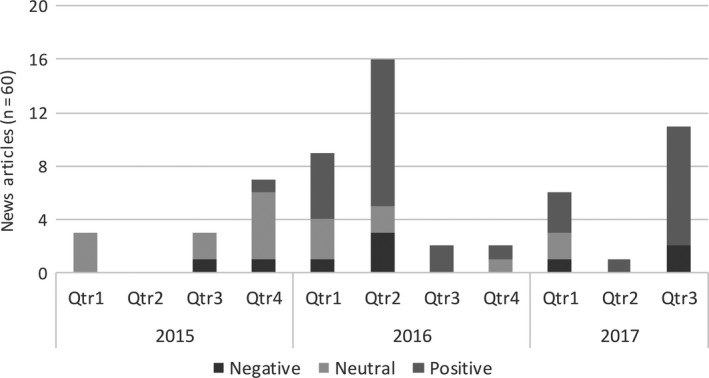
News stories 2015‐2016 (not weighted for influence)

Adding a temporal dimension to the analysis (see Figure 2) highlights the spike in media interest in organ donation that took place following the implementation of the Act in 2015, but also enables clear shifts in media tone towards the policy in Wales to be observed.

Previous research^9^, ^23^ studying news stories and organ donation has suggested a link between negative media coverage of organ donation and sensationalism—as represented by measures of deviance. We found no relationship in our data with 57% of articles coded as either “not at all unusual” or “somewhat unusual” in their content, while only 12% contained “extremely unusual” information. Social significance was greater with 60% of content coded as having moderate or major social significance. Combining the deviance and significance variables to create a measure of newsworthiness as suggested by Shoemaker and Cohen,^35^ we observe over the period of study a steady increase in the complexity of stories (Table 3), suggesting that coverage might have an increasing potential to influence decision making and agenda setting.

**Table 3:** Source classifications by year: valance and newsworthiness

Year	Number of sources	Valance			Deviance score^a^	Significance score^b^	Combined newsworthiness score^c^
		Negative	Neutral	Positive			
2015	13	2	10	1	1.77	2.31	4.08
2016	29	4	6	19	2.38	2.83	5.21
2017	18	3	2	13	2.56	2.67	5.22

a Mean deviance score.

b Mean significance score.

c Sum of deviance and significance scores.

Detailed examination of texts highlights a more complex picture beneath the headlines. Within the content, “organ donation” was mentioned 289 times with 64% of content coded as positive and only 7% as negative. We found almost no reports of comments that set out arguments against organ donation in principle, with media reflecting recent evidence of public opinion^36^ and the views of major UK faith groups.^37^ This contrasts with research where the media was observed to portray negative views of organ donors and transplants.^8^ Nonetheless, the tone of discussion within the studied content regarding the law change was less equivocal. While we only coded 6% of content as being negative towards the new law, we categorized 42% of coverage as neutral, and this was particularly noticeable in the first year studied.

Media coverage in 2015—when the Act was implemented—broadly summarized the preceding public debates that had taken place, with sceptical views presented alongside balancing statements from official (Welsh Government or NHS) sources:

…critics, including the Welsh Conservatives and the Church in Wales remain sceptical, arguing that the new system could be confusing, may alienate relatives of potential donors and even tarnish the image of organ donation (The Guardian 01/12/15)

Ministers have said more than two‐thirds of people in Wales are now aware of the changes and they hoped a “leap in consent rates” would follow (BBC News 01/12/15)

A number of news organizations were observed struggling to explain the change using unfamiliar terminology. The terms “soft opt‐out,” “presumed consent” and “deemed consent” were used frequently but not always contextualized or explained, and this lack of understanding led to some *negative* commentary while the implications of an organ donation register where people could both “opt‐out” and “opt‐in” were not fully understood. In particular, the notion of a “soft opt‐out” that retained a central role for families was not always presented:

Opt‐out organ donation will treat bodies like “clapped out cars” (Daily Telegraph 01/12/15)

Law that assumes you’re an organ donor mean body parts will be used unless person had already opted out (Daily Mail 20/11/15)

Throughout 2016 and into 2017, our analysis showed that the tone of coverage became more supportive of the Welsh policy. Whereas only 3% of news headlines in 2015 were positive, through 2016 and 2017 this increased to over 60%. We found that sensationalist language and inaccurate reporting declined as the reality of organ donation practice under the Act became clearer and evidence emerged of some success from the policy, for example “*Dozens saved” in six months by Welsh deemed consent organ donation system* (The Guardian, 14/06/2016).

A key message frame that emerged from the initial scoping of content was the notion of organ donation as a “gift” that is regarded as having wider societal benefit and driven by an individual's desire to help other people.^38^ In a number of cases, the law change was presented as restricting choice and taking away the ability of individuals to make a “gift” of an organ. Some commentators went so far as to reject the policy as paternalistic or even totalitarian:

Currently my family are aware that I have no objection to my organs being taken for the purposes of organ transplant but the moment the State decides it has control over my body and organs I will immediately opt out and inform my family in the strongest terms possible that they must refuse to give any consent to any removals as a matter of principle. I am happy to volunteer but I refuse absolutely to be bullied over the issue by arrogant medics and politicians (Daily Mail, 05/12/15)

Further criticism was reported by religious leaders as the policy was introduced:

“In the deeming of consent, there is always the risk that that idea of gift might vanish. From a Christian or moral perspective, I think the idea of giving someone a precious gift is something much better than others presuming that that gift can be eradicated and just taken.” The Rt Rev John Davies, Bishop of Swansea and Brecon (BBC News 01/12/15)

Previous research^39^ has found a relationship between a country's organ donation scheme and the way in which citizens perceive the act of donation. People in countries with opt‐in systems have been found to judge organ donation to be a far greater act of altruism than those in countries with an “opt‐out.” Within an opt‐in system, people who donate organs are exceptional. When people are presumed to be organ donors, the exceptional case is the individual reluctant to take part.^39^ It is unsurprising that during the policy transition period in Wales, such debates were played out. Over the three years studied, the potentially difficult change in narrative was seen to be modified in the media primarily through human interest stories that focused on the “gift of life” that was received as well as the gift of the organ by the donor:

“Every day I think of the selfless gift my donor and their family gave me” Woman describes how double lung transplant has changed her life (Wales Online 01/12/16)

Organ donation means Gwynedd Granddad has four kidneys after receiving gift of life… twice (The Daily Post 14/06/16)

The people of Wales who donate organs are potentially giving people the gift of life (ITV News 02/12/16)

Both locally and nationally, stories were published that illustrated either how being in Wales had led to lives being saved, or how *if* a person had been living in Wales, their life might have been saved. The changing way in which media framed this message was evident in the rise of positive mentions of organ donation “gift” within the new policy regime. Positive coverage rose from 43% in 2015 to 70% in 2016 alongside the wider positive view of Wales’ “opt‐out” position becoming the norm in media reporting both inside Wales and more widely across the UK:

**Tattoo on murdered teen's arm persuaded his mum to honour organ donor wish**… “I’m all for the new organ donation system, I think it opens up those questions around organ donation that perhaps people might not otherwise talk about.” (The Mirror 14/03/17)

**A family moved to Wales to increase son's chance of finding an organ donor**. Helen Lynch and her family moved to Pontypridd from Offaly in Ireland in December. Wales is the only part of the UK where people have to “opt out” of being an organ donor after a new law was introduced in 2015 (Wales Online 31/03/17)

A further topic considered in the literature and which appeared as an important message frame in the media was around the role of family within an “opt‐out” system. Study of this area^40^, ^41^ suggests that this is often an area of conflict of interest and control between potential donors, families, medical professionals and potential transplant recipients. In adopting a “soft opt‐out” system in Wales, families still have an important role in organ donation decisions, particularly where the deceased has not made their decision known, yet within the media, some reports have suggested that organs can be taken without the family's consent:

…the Welsh government had failed to explain the new system clearly, “including the fact that under the new arrangements family members do not have the right to stop organs being removed, even if it is against their wishes.” Darren Miller, Conservative Spokesman (The Guardian 01/12/15)

Nonetheless, our findings suggest a shift in tone over the study period. In 2015, 43% of news content discussing the role of families was negative falling to just 4% in 2017.

## 5 DISCUSSION

To set our findings within a policy context, consent rates rose from 48.5% in 2014/5 to 64% in May 2017. By 2018, the figure had risen to 72% equating to approximately 24.3 donors per million population, the highest amongst UK countries. However, with a relatively small numbers of cases each year an increase in the numbers of organ donors has yet to be observed.^42^ During the period 2015‐2017, we observed a change in valance of media content towards Wales’ organ donation policy, and on a number of levels. One aspect highlighted was the degree of similarity in content, stimulated by, and drawn primarily from official press releases. For example, in March 2016, Welsh Government published the following press release:

**New organ donation system has saved lives.** Fifteen people donated their organs in the first two months after a new soft opt‐out system for organ donation was introduced in Wales, new figures released today show.^43^

The press release was repeated with little editing in six news pieces with a verbatim quote from the Health and Social Services Minister used in each. Across all three years studied, we found a strong link between the number of news articles related to organ donation and official statements, raising questions of the intertextuality between media and the state. Such observations would suggest a one‐way relationship within a propaganda model^18^ contending that the state exerts a powerful influence over media reporting. Furthermore, as Curran et al^19^ suggest—and we observed in our findings—within this critical model, interdependency exists as the media relies on the state as a major source of “credible information” while the state is reliant on the media for the communication of its policies. However, our data also provide an indication of the opposing interpretation of the state‐media interaction; that the influence of the state is not absolute and that mass media organizations have a degree of autonomy and use this to influence policy setting.^19^ With some evidence that the Act had been successful in increasing consent rates in Wales, we observed how the tone of media coverage became more positive during 2016. A flurry of coverage in June, when new data seemed to indicate an increase in organ donations, can be seen as an important tipping point.

“Dozens saved” in six months by Welsh deemed consent organ donation system (The Guardian 14/06/16)

Half of organ transplants from deemed consent after new law (BBC News 14/06/16)

The idea that the new policy in Wales was successful was further embedded in a growing number of human interest stories regarding organ donation across the UK that cited the Welsh law change.

**Barrow mum saved by sister's kidney speaks about the importance of organ donation**… The former hairdresser is now a passionate advocate of organ donation following her operation in 2006. She is hoping the government will change the current opt‐in scheme to one similar to the one in Wales, where people must actively remove themselves from the organ donor list (The Cumbrian Mail 12/09/16)

The Welsh “success” was further used by UK media to highlight policy division and inconsistency across the other UK nations. Initially, Governments in both Scotland and England had been circumspect regarding changes to the organ donation status quo, preferring to wait for significant evidence from Wales. However, a number of media organizations began to appeal for change and in particular, The Mirror newspaper's “Change the Law for Life” campaign claimed that it was influential in Scotland's decision to consult and then in June 2017 to follow Wales in proposing an “opt‐out” system of consent to organ donation:

The Mirror’s campaign for a law change to boost organ donations and save lives has received a massive lift. Scotland has decided to follow Wales and bring in an opt‐out system, meaning more organs available for transplant (The Mirror 28/06/17)

A campaign for Wales' organ donation laws to be adopted in England has been launched by a mother whose daughter needed a transplant (BBC News 25/01/17)

The British Medical Association will lobby the three parliaments to follow the lead set by Wales, which in December introduced presumed consent for organ retrieval (The Guardian 22/06/16)

The UK Government subsequently followed Scotland by announcing a consultation in England on a “soft opt‐out” in December 2017 and supported a private members bill proposing “deemed consent” legislation in March 2018. This might be perceived as representing a major change in culture and public opinion towards organ donation. Across time, we found fewer dissenting opinions in the media although it is unclear whether this is genuinely representative of societal pressures or represents a “negotiation of hegemony”^20^ between the state and the media. Interestingly, in interviews with non‐donor relatives other research^44^ has found that some family members feel pressurized by media to “give the gift of life” resulting in feelings of guilt and selfishness when they choose not to.

## 6 CONCLUSION

Media content provides a window into changing public discourse and political agenda setting related to the issue of organ donation consent systems. Looking at content over time allows for a nuanced understanding of the relationship between policy makers, implementing institutions, and the public, while also analysing the ways in which each party shapes press coverage.

Our analysis charts an observable change in tone and view towards deemed consent organ donation with a public health communication campaign successfully setting the agenda and informing the debate. Over the three years of study, there is evidence that the principle of deemed consent and the “soft opt‐out” system has become increasingly newsworthy, portrayed in the media as a public good, even with limited evidence of actual impact. Furthermore, discussion of the wider moral and ethical issues that dominated coverage prior to implementation has largely disappeared.

If, as the literature suggests, the media both reflects and influences public opinion, then we see in the evidence a change in attitude towards the Human Transplantation (Wales) Act 2013 and more importantly, towards organ donation itself. However, content analysis is only the very first, preliminary step in the investigation of changing public perceptions and attitudes. While the media has a central role in communicating to the public, people do not absorb messages uncritically and further research is needed to confirm these findings, and more critically to investigate whether changes in opinion translate into behavioural change that result in increases in organ donations.
